# A Retrospective Study of Respiratory Viruses in a Four-Year Study of Nasal Swabs from Patients with Severe Influenza-like Symptoms in the Lazio Region, Italy

**DOI:** 10.3390/v17030452

**Published:** 2025-03-20

**Authors:** Giuseppe Sberna, Licia Bordi, Cosmina Mija, Enrico Girardi, Fabrizio Maggi, Eleonora Lalle

**Affiliations:** 1 Laboratory of Virology and Biosafety Laboratories, National Institute for Infectious Diseases Lazzaro Spallanzani—IRCCS, 00149 Rome, Italy; 2 Scientific Direction, National Institute for Infectious Diseases Lazzaro Spallanzani—IRCCS, 00149 Rome, Italy

**Keywords:** respiratory viruses, SARS-CoV-2, post-pandemic era

## Abstract

The global outbreak of severe acute respiratory syndrome-related coronavirus 2 (SARS-CoV-2) and the strategies adopted by different nations have affected and altered the transmission of different respiratory pathogens around the world. We examined the impact of SARS-CoV-2 on the spread of respiratory viruses in the period between 2021 and 2024 in patients with severe influenza-like symptoms in the Lazio region using multiplex PCR tests for the identification of common seasonal respiratory viruses. Our data reveal a change in the transmission of respiratory viruses from 2021 to 2024, with a sharp decline in the transmission of SARS-CoV-2 and a rise in the transmission of other respiratory viruses, especially influenza viruses, and human rhinovirus/enterovirus in 2024. Moreover, viral co-infections, both those involving two viruses and those involving three viruses, have also increased. This work shows how the spread of SARS-CoV-2 influenced the spread of other respiratory viruses over four years in patients with severe influenza-like symptoms in the Lazio region. In conclusion, the resurgence and fluctuation of various respiratory viruses emphasize the dynamic nature of viral epidemiology in the post-pandemic context and highlight the ongoing need for vigilant public health monitoring and intervention strategies.

## 1. Introduction

The Coronavirus disease 19 (COVID-19) pandemic has left a wound that will be forever etched on the world’s population [1]. In addition, the rapid spread of severe acute respiratory syndrome-related coronavirus 2 (SARS-CoV-2) and the preventive strategies adopted by different nations have influenced and changed the spread of respiratory pathogens around the world [2,3,4,5]; Italy has also undergone these changes [6,7]. For example, at the beginning of the pandemic, in the Lazio region, respiratory viruses seemed to circulate without being affected by SARS-CoV-2, when comparing the sixth to the fifteenth weeks of 2019–2020 [8]. On the contrary, a change in the circulation of respiratory viruses was observed when analyzing weeks 41–50 of 2019, 2020, and 2021; in particular, an increase in positive samples for the human orthopneumovirus, commonly known as human respiratory syncytial virus (HRSV) and human rhinovirus/enterovirus (HRV/EV), was detected [9]. Moreover, during the 2022–2023 influenza season, 7.96% of viral co-infections were observed in SARS-CoV-2-positive nasopharyngeal swabs (NPS) [10]. The epidemiological impact of viral respiratory mono- and co-infections in Italy has been notable, especially among vulnerable groups. Surveillance data show that advanced age, chronic diseases such as cardiovascular disease, chronic obstructive pulmonary disease (COPD), diabetes, and immunosuppressive conditions are major risk factors for severe outcomes in both pre- and post-pandemic periods [11,12]. The European Respiratory Virus Surveillance Summary (ERVISS) provides integrated epidemiological data on the circulation of respiratory viruses, highlighting the increased morbidity and mortality associated with these infections [13]. During the post-pandemic period, the prevalence of co-infections has been observed, with older adults and individuals with chronic conditions being disproportionately affected [14]. Understanding these dynamics is important for developing targeted public health interventions and improving patient outcomes.

Additionally, the role of acquired immunity due to natural infection or vaccination has significantly influenced SARS-CoV-2 transmission patterns between 2022 and 2023. A substantial proportion of the population had either been vaccinated or developed immunity from previous infections, which played a crucial role in shaping the epidemiological landscape. Studies show that natural immunity and vaccination both provide strong protection against severe disease, hospitalization, and death, although the level and duration of protection can vary [15,16]. This acquired immunity has contributed to changes in the circulation of SARS-CoV-2 and other respiratory viruses, underscoring the importance of vaccination campaigns and natural immunity in managing the pandemic and its aftermath.

This study examines the impact of the SARS-CoV-2 pandemic on the circulation of respiratory viruses in the Lazio region (Italy). It investigates how COVID-19 and the non-pharmaceutical countermeasures have affected the spread of these viruses. The main objectives were to analyze the influence of SARS-CoV-2 on the circulation of respiratory viruses from 2021 to 2024 in patients with severe flu-like symptoms and to monitor the trend of viral co-infections.

## 2. Materials and Methods

### 2.1. Clinical Specimens and Patients

NPS samples collected from patients with severe influenza-like symptoms admitted to various hospitals in the Lazio region (Italy) were sent to the Laboratory of Virology and Biosafety Laboratories of the National Institute for Infectious Diseases Lazzaro Spallanzani in Rome for routine virological analyses from 1 January 2021 to 31 December 2024. This population primarily consisted of hospitalized patients with severe diseases, reflecting a high burden of illness and significant healthcare needs. Table 1 shows the demographic characteristics of patients.

The population studied consisted of 5082 patients, with a median age of 62 years. The gender distribution was 42% female and 58% male. Most patients were elderly, with a significant proportion aged 15 years and older, particularly in the first semesters of 2022 and 2023, where the median ages reached 70 and 69 years, respectively. Information regarding the hospitalization department was obtained for 35% of the analyzed patients (Table 2).

Overall, the population under investigation represents a vulnerable group with severe influenza-like symptoms, requiring comprehensive medical attention and highlighting the importance of targeted public health interventions to manage and mitigate the impact of respiratory infections in such high-risk individuals.

### 2.2. Molecular Assays

Samples were tested for qualitative detection and differentiation of respiratory viruses and bacteria using two multiplex PCR assays, QIAstat-Dx Respiratory SARS-CoV-2 Panel (Qiagen s.r.l., Germantown, MD, USA [17]) or BIOFIRE^®^ Respiratory Panel 2.1 plus (BioFire Diagnostics LLC, Salt Lake, UT, USA [18]), based on availability in the laboratory.

QIAstat-Dx Respiratory SARS-CoV-2 Panel is able to detect and differentiate 21 respiratory targets (18 viruses and 3 bacteria), among which were SARS-CoV-2 and the following other viral pathogens: human adenovirus (HAdV), human alphacoronavirus 229E, human betacoronavirus HKU1, human alphacoronavirus NL63, human betacoronavirus OC43, human metapneumovirus A + B (HMPV), Influenza A virus (FLUAV; including FLUAV-H1, FLUAV-H3, FLUAV-H1N1pdm09), Influenza B virus (FLUBV), human parainfluenza virus (HPIV; including HPIV-1, HPIV-2, HPIV-3, HPIV-4), HRV/EV, and HRSV A + B.

BIOFIRE^®^ Respiratory Panel 2.1 plus targeted a total of 23 respiratory pathogens, including 19 viruses and 4 bacteria. Therefore, both assays can detect the same viruses, with BIOFIRE^®^ Respiratory Panel 2.1 plus also being able to identify the Middle East respiratory syndrome-related coronavirus. Both assays were performed according to the manufacturers’ instructions.

### 2.3. Statistical Analysis

Data management was performed using GraphPad Prism version 10.00 (GraphPad Software, La Jolla, CA, USA [19]). Data were analyzed in six-month periods using a chi-square test; a *p*-value < 0.05 was considered significant.

## 3. Results

From 1 January 2021 to 31 December 2024, a total of 5082 NPS were tested, among which 2398 (47%) resulted in being positive for viral detection, but with a statistically significant yearly variation (*p* < 0.0001; Chi-square test). Table 3 shows that the highest detection frequency was observed in 2021 (53% positive swabs) and the lowest was observed in 2024 (43% positive specimens).

**Figure 1:**
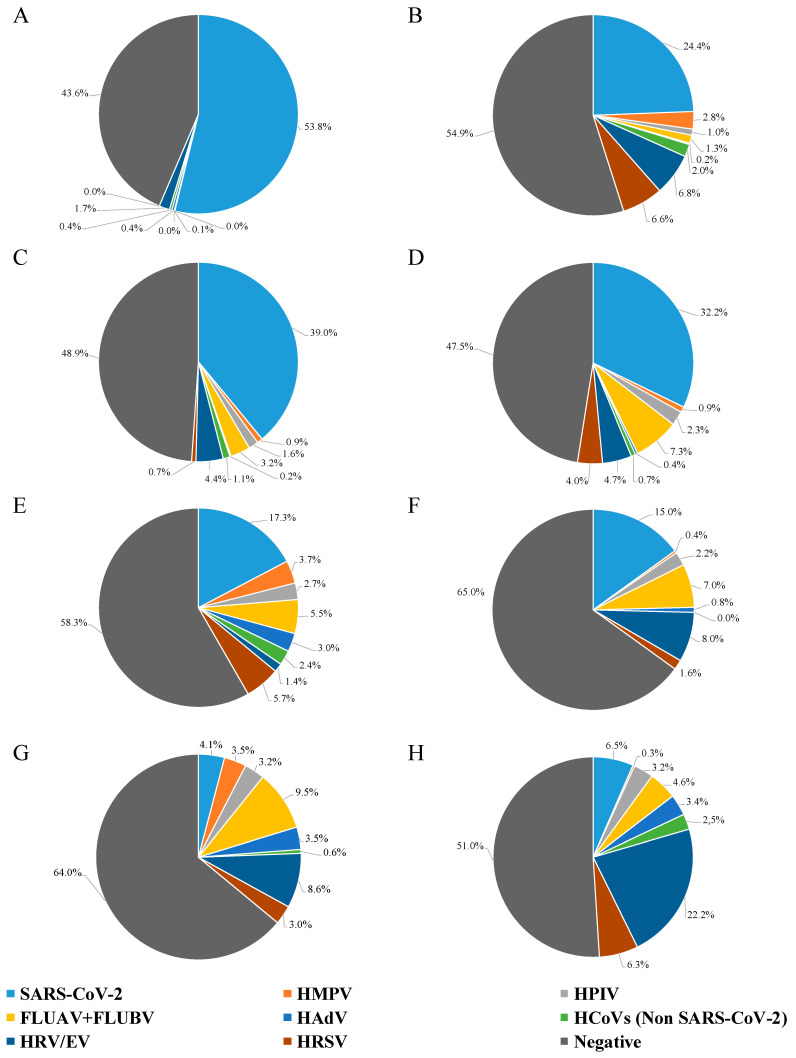
Results of NPS testing from hospitalized patients with severe influenza-like symptoms, grouped every six months. (**A**) NPSs tested from January to June 2021; (**B**) NPSs tested from July to December 2021; (**C**) NPSs tested from January to June 2022; (**D**) NPSs tested from July to December 2022; (**E**) NPSs tested from January to June 2023; (**F**) NPSs tested from July to December 2023; (**G**) NPSs tested from January to June 2024; and (**H**) NPSs tested from July to December 2024.

Figure 1 shows the prevalence of different viruses, grouped into six-month intervals every year. In more detail, in the first half of 2021, SARS-CoV-2 was present in 54% of the NPS, while HAdV, HPIV, non-SARS-CoV-2 human coronavirus (HCoV), and HRV/EV were detected in less than 2.0% of the samples (0.4%, 0.1%, 0.4%, and 1.7%, respectively) (Figure 1A). By the second half of 2021, a shift was evident (Figure 1B), with SARS-CoV-2 being at 24% prevalence, HRV/EV at 6.8%, HRSV at 6.6%, and the remaining viruses being at variable prevalence, under 2.8% (range: 0.2–2.8%).

There was no difference in the detection of SARS-CoV-2 between the first half (39%) and the second half (32%) of 2022; likewise, HMPV, HAdV, non-SARS-CoV-2 HCoV, and HRV/EV had similar detection rates (Figure 1C,D). For HPIV and FLUAV + FLUBV, a two-fold increase in the detection rate was observed between the first and the second half of 2022 (Figure 1C,D), with HPIV remaining stable until 2024 (about 2.5%; Figure 1D–G). Noteworthy, HRSV had a nearly six-fold increase in detection rate from the first to the second half of 2022 (0.7% to 4.0%; Figure 1C,D).

Looking at 2023, the SARS-CoV-2 detection rate had a substantial decrease both in the first and the second semester for 2021 and 2022 (less than 18%; Figure 1, Panels E, and F). Non-SARS-CoV-2 HCoV, HAdV, and HMPV had a percentage of about 3.0% in the first half of 2023, decreasing below 1.0% in the second half of 2023. The percentage change between the first and second half of 2023 was 5.7% to 1.6% for HRSV and 5.5% to 7.0% for FLUAV + FLUBV; moreover, there was a notable rise (from 1.4% to 8.0%) in HRV/EV-positive samples.

In the first half of 2024, the situation completely changed (Figure 1G): FLUAV + FLUBV were the most frequently detected (9.5%), followed by HRV/EV (8.6%); SARS-CoV-2 dropped sharply, being retrieved only from about 4.0% of samples; HMPV, HRSV, HPIV, and HAdV were present in about 3.0%, while non-SARS-CoV-2 HCoV almost vanished, being detected in only 0.6% of cases. In the last six months of 2024 (Figure 1H), HRV/EV were found in 22.2% of the samples, while SARS-CoV-2 and HRSV were the second and third most frequently detected viruses, with very similar detection rates of 6.5% and 6.3%, respectively. FLUAV + FLUBV were identified in 4.6% of the samples, whereas HPIV and HAdV remained stable compared to the previous semester. Non-SARS-CoV-2 HCoV increased to 2.5%, while HMPV dropped drastically to 0.3%. Notably, the variations in frequency for each individual analyzed pathogen was significantly different for all of them over time (*p* < 0.0001; Chi-square test).

**Figure 2:**
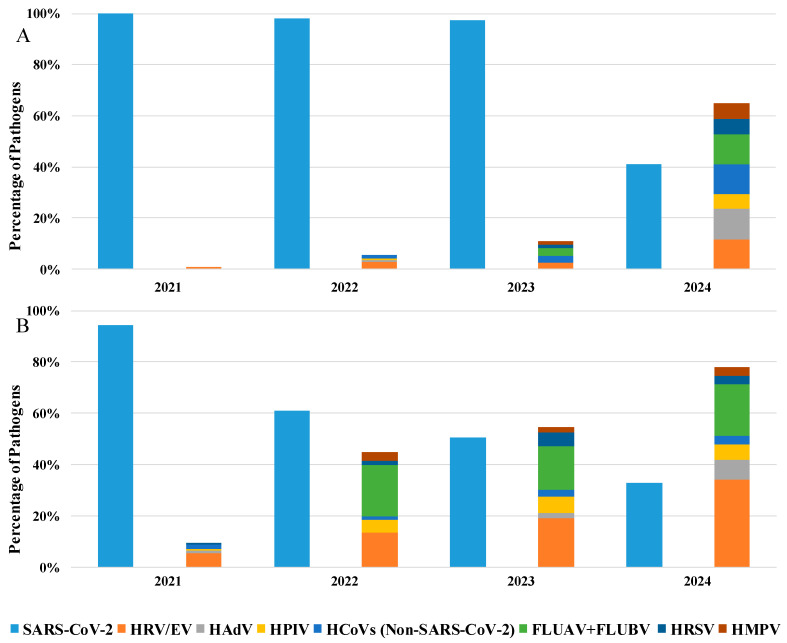
Percentage of detected pathogens in positive samples of patients hospitalized in the (**A**) General Hospital Ward and (**B**) in Intensive Care Unit from 2021 to 2024.

Furthermore, as shown in Table 2, details about the hospitalization department were available for 35% of the analyzed patients: among these, 76% were in a GHW, while 24% were in the ICU. Specifically, analyzing the positive samples from patients hospitalized in GHW (Figure 2A), SARS-CoV-2 remains the predominant pathogen until 2023 (51%), but an increase in the presence of other respiratory viruses was evident starting from 2022 (45%) to 2024 (78%), where HRV/EV was the most commonly detected pathogen (34%), followed SARS-CoV-2 (32%).

Moreover, also in ICU patients, the predominant detected pathogen was SARS-CoV-2, which remains satiable until 2023 (97%), with a mild presence of other respiratory viruses. Otherwise, in 2024, we observe a substantial decrease in SARS-CoV-2 detection (41%) with the reappearance of other respiratory viruses (65%; Figure 2B).

**Figure 3:**
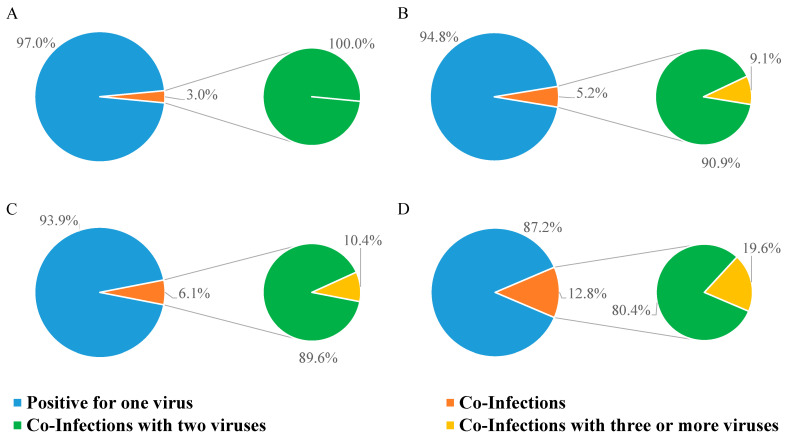
Percentage of NPSs testing positive for one, two, or three viruses. (**A**) Positive NPSs in 2021; (**B**) positive NPSs in 2022; (**C**) positive NPSs in 2023; (**D**) positive NPSs in 2024.

We also checked the frequency of viral co-infections (Figure 3). As shown in Table 4, most co-infections were found in GHW in each year analyzed in the study (Table 4).

In 2021, only 3.0% of positive samples had double infections (Figure 3A); in 2022, the rate of multiple infections rose to 5.2%, 9.1% of which were triple infections (Figure 3B); in 2023, the percentage of samples with multiple infections was 6.1%, among which, 10.4% were triple infections (Figure 3C); multiple infections kept increasing in 2024 (12.8%), and of these, 19.6% were triple infections (Figure 3D).

As shown in Table 5, SARS-CoV-2 and HRV/EV are the most frequently involved pathogens in co-infections for the years 2021 (57.9% and 57.9%, respectively), 2022 (68.8% and 37.5%, respectively), and 2023 (40.0% and 56.0%, respectively), while, in 2024, a clear decrease in the percentage of SARS-CoV-2 (13.9%) and a marked prevalence of HRV/EV (80.2%) was observed. The other viruses fluctuate with comparable values over the years, except for HAdV, which was not detected in the co-infections of 2021 and 2022, reappearing in co-infections in 2023 (12.0%) and increasing in 2024 (31.4%; Table 5).

The detection rate of co-infections was significantly different over time (*p* < 0.0001; Chi-square test), as well as for the detection rate of co-infections with three or more viruses (*p* = 0.0078; Chi-square test).

## 4. Discussion

The pandemic caused by SARS-CoV-2 resulted in a temporary disappearance of seasonal respiratory viruses [5,6,20,21], mainly due to preventive measures such as isolation, social distancing, and mask use [2]. However, after the pandemic, respiratory viruses reappeared and took over the space that was previously occupied by SARS-CoV-2 [3]. In more detail, this study shows a considerable drop in the detection rate of SARS-CoV-2 in the NPSs of patients with severe influenza-like symptoms, decreasing from 54% in the first half of 2021 to 6.5% in the last six months of 2024 and a parallel increase for other respiratory viruses. Our results show the almost complete absence of non-SARS-CoV-2 respiratory infections during the first half of 2021, reflecting what has been reported in different parts of Italy and the world [22,23]. A gradual and steady increase in HRV/EV were shown from 2021 (1.7%) to 2024 (22.2%), being the current most widely detected virus and showing a fluctuating trend in previous years. FLUAV + FLUBV were among the most frequently detected viruses in the first half of 2024 (9.5%), in contrast to 2021, when they had completely disappeared. HRSV, HMPV, non-SARS-CoV-2 HCoV, HPIV, and HAdV displayed variable trends over the years. For example, HRSV, in 2024, showed a lower detection rate in the first half of this year (3.0%), which increased in the second half (6.3%). A similar detection rate was observed in 2021 (rising from 0.0% to 6.6%) and in 2022 (increasing from 0.7% to 4.0%). However, this trend was not evident in 2023, when HRSV was detected more frequently in the first half of this year (5.7%) than in respect to the second half (1.6%). HMPV showed a similar trend between 2023 and 2024, differing from its behaviour in 2021 and 2022. Furthermore, non-SARS-CoV-2 HCoV, which almost disappeared (0.6%) in the first half of 2024, reappeared in 2.5% of cases in the second half of this year. This detection rate appears to be comparable to 2021, but not to 2022 or 2023. This scenario was also evident from the results obtained by stratifying patients between GHW and ICU. In fact, in regular GHW, the presence of non-SARS-CoV-2 respiratory viruses began in 2021 (10%) and increased over the years (78% in 2024), reflecting a resurgence in the circulation of these pathogens coinciding with the reduction in pandemic containment measures and the introduction of the COVID-19 vaccination campaign. On the other hand, in ICU, SARS-CoV-2 remained the predominant pathogen until 2023 (97%), while other respiratory viruses were poorly represented (11%). A reversal was observed only in 2024, when the presence of SARS-CoV-2 decreased to 41%, with a resurgence in the circulation of other respiratory viruses (65%), highlighting that, despite the introduction of the anti-COVID-19 vaccination, SARS-CoV-2 remains the main etiological agent of respiratory diseases in ICU.

Overall, our analysis confirms that non-pharmaceutical countermeasures (i.e., isolation, social distancing, and mask use) had an impact on the seasonal trends of many respiratory viruses, with SARS-CoV-2 emerging as the predominant circulating mono-infection. The situation changed with the easing of containment measures and the initiation of the COVID-19 vaccination campaign; gradually over time, the circulation of other respiratory viruses resumed, increasing from 2.6% in 2021 to 31.9% in 2024. Similar data were obtained in other studies, including those conducted outside of Italy, confirming that, during the post-pandemic period, the circulation of many respiratory viruses was restored [24,25].

Finally, considering viral co-infections, it should also be emphasized how the percentage of co-infections started at 3% in 2021 and reached 12.8% in 2024, with a higher prevalence in GHW patients than in ICU patients. In more detail, SARS-CoV-2 and HRV/EV were the most common pathogens in co-infections during 2021, 2022, and 2023. However, in 2024, SARS-CoV-2 significantly decreased to 13.9%, while HRV/EV’s prevalence increased to 80.2%. Other viruses remained stable over the years, except for HAdV, which was absent in 2021 and 2022 co-infections, but reappeared in 2023 (12.0%) and increased further in 2024 (31.4%). Changes in respiratory virus spread could also be attributed to viral interference, a phenomenon for which an initial exposure to a virus, causing an active viral infection and spreading within the human respiratory system, makes most cells temporarily non-susceptible to subsequent infections [26,27,28]. Viral interference requires close viral exposure, meaning that all viruses need to share similar ecological conditions (such as a cold climate). At the population level, viral interference manifests as an ecological phenomenon where an epidemic caused by one virus delays the onset or hastens the conclusion of an epidemic caused by another virus [26]. Although these episodes are difficult to demonstrate, the present findings could also find a possible explanation through this phenomenon.

From this study, monitoring mono- and co-infections is crucial for understanding the dynamics of infectious diseases and their impact on public health. By tracking these infections, we can identify patterns of co-circulating pathogens and their potential interactions, which may influence disease severity and treatment outcomes. These data are essential for developing targeted preventive strategies, such as vaccination campaigns or enhanced surveillance. Additionally, monitoring infections helps inform clinical decision-making, enabling healthcare professionals to provide more accurate diagnoses and personalized treatment plans. Overall, continuous surveillance is key to improving patient care and controlling the spread of infections.

## 5. Limitations of This Study

This study, while providing valuable insights into the trends of respiratory viruses over four years in the Lazio region, has several limitations. Its retrospective design may introduce biases related to data collection and patient selection, and the regional focus limits the generalizability of the findings to other areas. Additionally, the lack of detailed information on patient comorbidities, outcomes, and vaccination status, as well as potential confounding factors related to COVID-19 measures, further limits the study. Lastly, the absence of data on the severity of COVID-19 cases could influence the interpretation of the impact of SARS-CoV-2 on the circulation of other respiratory viruses. These limitations suggest the need for cautious interpretation of the findings and highlight areas for future research, such as examining whether patients with persistent SARS-CoV-2 infections are more susceptible to coinfections with other respiratory viruses, experience increased disease severity, have higher rates of coinfections, or face poorer prognoses [29,30,31].

## 6. Conclusions

This study shows how SARS-CoV-2 changed the circulation of respiratory viruses in the post-pandemic era in patients with severe influenza-like symptoms in the Lazio region. Importantly, this resurgence and fluctuation in various respiratory viruses emphasizes the dynamic nature of viral epidemiology in the post-pandemic context and highlights the ongoing need for vigilant public health monitoring and intervention strategies.

## Figures and Tables

**Table 1:** Demographic characteristics of patients with severe influenza-like symptoms included in the study. * 95% CI: 95% of confidence interval.

Semester	Examined			
	Total *n*.	*n*. Female (%)	*n*. Male (%)	Median Age (95% CI) *
1° 2021	913	366 (40)	547 (60)	64 (62–66)
2° 2021	466	192 (41)	274 (59)	57 (53–59)
1° 2022	432	178 (41)	254 (59)	70 (68–72)
2° 2022	423	184 (43)	239 (57)	69 (65–70)
1° 2023	666	294 (44)	372 (56)	69 (67–71)
2° 2023	490	213 (43)	277 (57)	58 (56–61)
1° 2024	640	240 (37)	400 (63)	56 (53–60)
2° 2024	1052	451 (43)	601 (57)	49 (46–52)
Total	5082	2118 (42)	2964 (58)	61 (60–62)

**Table 2:** Information regarding the hospitalization department of patients. * ICU: Intensive Care Unit; ^#^ GHW: General Hospital Ward.

Year	Total *n*.	Patients *n*. with Information (%)	Department	Patients *n*. (%)
2021	1379	478 (35)	ICU *	135 (28)
			GHW ^#^	343 (72)
2022	855	377 (44)	ICU	162 (43)
			GHW	215 (57)
2023	1156	464 (40)	ICU	90 (19)
			GHW	374 (81)
2024	1692	467 (28)	ICU	43 (9)
			GHW	424 (91)
Total	5082	1786 (35)	ICU	430 (24)
			GHW	1356 (76)

**Table 3:** Molecular testing of 5082 NPS from patients with severe influenza-like symptoms hospitalized between 1 January 2021 and 31 December 2024. ^a^ Significantly different from the years 2023 to 2024 at *p* < 0.0001 and *p* < 0.0001, respectively (Chi-square test); ^b^ significantly different from the years 2023 to 2024 at *p* < 0.001 and *p* < 0.0001, respectively (Chi-square test).

Year	Examined	*n*. Positive (%)
2021	1379	727 (53) ^a^
2022	855	445 (52) ^b^
2023	1156	506 (44)
2024	1692	720 (43)
Total	5082	2398 (47)

**Table 4:** Prevalence of co-infections in Intensive Care Unit (ICU) patients compared to the General Hospital Ward (GHW).

Years	GHW	ICU
2021	80.0%	20.0%
2022	58.3%	41.7%
2023	66.7%	33.3%
2024	93.7%	6.3%

**Table 5:** Detection rate of pathogens involved in co-infection between 1 January 2021 and 31 December 2024.

	2021	2022	2023	2024
SARS-CoV-2	57.9%	68.8%	40.0%	13.9%
HRV/EV	57.9%	37.5%	56.0%	80.2%
HPIV	10.5%	12.5%	12.0%	22.1%
FLUAV + FLUBV	21.0%	25.0%	12.0%	19.8%
HAdV	0.0%	0.0%	12.0%	31.4%
HCoV (non-SARS-CoV-2)	15.8%	25.0%	20.0%	16.3%
HMPV	15.8%	12.5%	24.0%	8.1%
HRSV	21.0%	18.8%	32.0%	30.2%

## Data Availability

The original contributions presented in the study are included in the article; further inquiries can be directed to the corresponding author.

## References

[1] Ammar A., Trabelsi K., Brach M., Chtourou H., Boukhris O., Masmoudi L., Bouaziz B., Bentlage E., How D., Ahmed M. (2021). Effects of home confinement on mental health and lifestyle behaviours during the COVID-19 outbreak: Insights from the ECLB-COVID-19 multicentre study. Biol. Sport.

[2] Dipartimento Della Protezione Civile—Governo Italiano. https://www.protezionecivile.gov.it/it/normativa/dpcm-del-13-ottobre-2020-sulle-misure-di-contrasto-e-contenimento-dell-emergenza-covid-19/.

[3] Luštrek M., Cesar Z., Suljič A., Kogoj R., Knap N., Virant M.J., Uršič T., Petrovec M., Avšič-Županc T., Korva M. (2024). Influenza A, Influenza B, human respiratory syncytial virus and SARS-CoV-2 molecular diagnostics and epidemiology in the post COVID-19 era. Respir. Res..

[4] Fukuda Y., Togashi A., Hirakawa S., Yamamoto M., Fukumura S., Nawa T., Honjo S., Kunizaki J., Nishino K., Tanaka T. (2023). Resurgence of human metapneumovirus infection and influenza after three seasons of inactivity in the post-COVID-19 era in Hokkaido, Japan, 2022–2023. J. Med. Virol..

[5] Chow E.J., Uyeki T.M., Chu H.Y. (2022). The effects of the COVID-19 pandemic on community respiratory virus activity. Nat. Rev. Microbiol..

[6] Palmas G., Trapani S., Agosti M., Alberti I., Aricò M., Azzari C., Bresesti I., Bressan S., Caselli D., Cazzato S. (2024). Associazione Ospedali Pediatrici Italiani (AOPI) Network. Disrupted Seasonality of Respiratory Viruses: Retrospective Analysis of Pediatric Hospitalizations in Italy from 2019 to 2023. J. Pediatr..

[7] Sclavi L., Bertelli M., Messali S., Caruso A., Caccuri F. (2024). Epidemiology and molecular characterization of respiratory viruses at the end of COVID-19 pandemic in Lombardy, Northern Italy. New Microbiol..

[8] Sberna G., Amendola A., Valli M.B., Carletti F., Capobianchi M.R., Bordi L., Lalle E. (2020). Trend of respiratory pathogens during the COVID-19 epidemic. J. Clin. Virol..

[9] Sberna G., Lalle E., Valli M.B., Bordi L., Garbuglia A.R., Amendola A. (2022). Changes in the Circulation of Common Respiratory Pathogens among Hospitalized Patients with Influenza-like Illnesses in the Lazio Region (Italy) during Fall Season of the Past Three Years. Int. J. Environ. Res. Public Health.

[10] Bordi L., Vulcano A., Sberna G., Nonis M., Giacomini P., Maggi F., Fontana C., Lalle E. (2023). Co-Circulation of SARS-CoV-2 and Other Respiratory Pathogens in Upper and Lower Respiratory Tracts during Influenza Season 2022–2023 in Lazio Region. Microorganisms.

[11] Santus P., Danzo F., Signorello J.C., Rizzo A., Gori A., Antinori S., Gismondo M.R., Branbilla A.M., Contoli M., Rizzardini G. (2024). Burden and risk factors for coinfections in patients with a viral respiratory tract infection. Pathogens.

[12] Mauro M.V., Greco S., Pellegrini M., Campagna T., Caprino F., Elia N., Mastroianni A., Greco F. (2024). Epidemiology and clinical impact of single and multi-viral respiratory infections in post-pandemic era. New Microbiol..

[13] The European Respiratory Virus Surveillance Summary (ERVISS). 25 October 2023. https://www.ecdc.europa.eu/en/publications-data/european-respiratory-virus-surveillance-summary-erviss.

[14] European Centre for Disease Prevention and Control Acute Respiratory Infections in the EU/EEA: Epidemiological Update and Current Public Health Recommendations. https://www.ecdc.europa.eu/en/news-events/acute-respiratory-infections-eueea-epidemiological-update-and-current-public-health.

[15] Stein C., Nassereldine H., Sorensen R.J.D., Amlag J.O., Bisignano C., Byrne S., Castro E., Coberly K., Collins J.K., Dalos J. (2023). Past SARS-CoV-2 infection protection against re-infection: A systematic review and meta-analysis. Lancet.

[16] Tu W., Zhang P., Roberts A., Allen K.S., Williams J., Embi P., Grannis S. (2023). SARS-CoV-2 Infection, Hospitalization, and Death in Vaccinated and Infected Individuals by Age Groups in Indiana, 2021–2022. Am. J. Public Health.

[17] Qiagen. https://www.qiagen.com/us/products/diagnostics-and-clinical-research/infectious-disease/qiastat-dx-syndromic-testing/qiastat-dx-na.

[18] Biomerieux. https://www.biomerieux.com/corp/en/our-offer/clinical-products/biofire-respiratory-2-1-panels.html#tabs-8cd7ba93fe-item-51e9655c24.

[19] GraphPad. https://www.graphpad.com/.

[20] Mansuy J.M., Bourcier M., Trémeaux P., Dimeglio C., Izopet J. (2021). COVID-19 pandemic period, where are the seasonal viruses?. J. Med. Virol..

[21] Tang J.W., Bialasiewicz S., Dwyer D.E., Dilcher M., Tellier R., Taylor J., Hua H., Jennings L., Kok J., Levy A. (2021). Where have all the viruses gone? Disappearance of seasonal respiratory viruses during the COVID-19 pandemic. J. Med. Virol..

[22] De Maio F., Fiori B., Bianco D.M., Sanguinetti M., Sali M. (2023). Respiratory viruses in the pre and post-pandemic periods in an Italian tertiary hospital. Immun. Inflamm. Dis..

[23] Wang H., Zheng Y., de Jonge M.I., Wang R., Verhagen L.M., Chen Y., Li L., Xu Z., Wang W. (2022). Lockdown measures during the COVID-19 pandemic strongly impacted the circulation of respiratory pathogens in Southern China. Sci. Rep..

[24] Cho H.J., Rhee J.E., Kang D., Choi E.H., Lee N.J., Woo S., Lee J., Lee S.W., Kim E.J., Yun K.W. (2024). Epidemiology of Respiratory Viruses in Korean Children Before and After the COVID-19 Pandemic: A Prospective Study from National Surveillance System. J. Korean Med. Sci..

[25] Jia W., Zhang X., Sun R., Li P., Zhen X., Li Y., Wang D., Li C., Song C. (2024). Changes in the epidemiological characteristics of influenza in children in Zhengzhou, China, in the post-COVID-19 era. J. Korean Med. Sci..

[26] Piret J., Boivin G. (2022). Viral Interference between Respiratory Viruses. Emerg. Infect. Dis..

[27] Waterlow N.R., Flasche S., Minter A., Eggo R.M. (2021). Competition between RSV and influenza: Limits of modelling inference from surveillance data. Epidemics.

[28] Wu A., Mihaylova V.T., Landry M.L., Foxman E.F. (2020). Interference between rhinovirus and influenza A virus: A clinical data analysis and experimental infection study. Lancet Microbe.

[29] Machkovech H.M., Hahn A.M., Wang J.G., Grubaugh N.D., Halfmann P.J., Johnson M.C., Lemieux J.E., O’Connor D.H., Piantadosi A., Wei W. (2024). Persistent SARS-CoV-2 infection: Significance and implications. Lancet Infect. Dis..

[30] Pavia G., Quirino A., Marascio N., Veneziano C., Longhini F., Bruni A., Garofalo E., Pantanella M., Manno M., Gigliotti S. (2024). Persistence of SARS-CoV-2 infection and viral intra- and inter-host evolution in COVID-19 hospitalized patients. J. Med. Virol..

[31] Babawale P.I., Guerrero-Plata A. (2024). Respiratory Viral Coinfections: Insights into Epidemiology, Immune Response, Pathology, and Clinical Outcomes. Pathogens.

